# Genome Sequences of SARS-CoV-2 P.1 (Variant of Concern) and P.2 (Variant of Interest) Identified in Uruguay

**DOI:** 10.1128/MRA.00410-21

**Published:** 2021-05-27

**Authors:** Yanina Panzera, Natalia Goñi, Lucía Calleros, Natalia Ramos, Sandra Frabasile, Ana Marandino, Gonzalo Tomás, Claudia Techera, Sofía Grecco, Eddie Fuques, Viviana Ramas, Leticia Coppola, María Rosa Flieller, Noelia Morel, María Noel Cortinas, Cristina Mogdasy, Juan Arbiza, Adriana Delfraro, Ruben Pérez, Héctor Chiparelli

**Affiliations:** a Sección Genética Evolutiva, Departamento de Biología Animal, Instituto de Biología, Facultad de Ciencias, Universidad de la República, Montevideo, Uruguay; b Centro Nacional de Referencia de Influenza y otros Virus Respiratorios, Departamento de Laboratorios de Salud Pública, Ministerio de Salud Pública, Montevideo, Uruguay; c Sección Virología, Instituto de Biología e Instituto de Química Biológica, Facultad de Ciencias, Universidad de la República, Montevideo, Uruguay; DOE Joint Genome Institute

## Abstract

Two severe acute respiratory syndrome coronavirus 2 (SARS-CoV-2) variants associated with increased transmission and immune evasion, P.1 and P.2, emerged in Brazil and spread throughout South America. Here, we report genomes corresponding to these variants that were recently detected in Uruguay. These P.1 and P.2 genomes share all substitutions that are characteristic of these variants.

## ANNOUNCEMENT

Severe acute respiratory syndrome coronavirus 2 (SARS-CoV-2) is a novel member of the genus *Betacoronavirus* (family *Coronaviridae*) and the causative agent of the ongoing coronavirus disease 2019 (COVID-19) pandemic (1). The analysis of the SARS-CoV-2 RNA genome has been crucial for understanding its origin and spread and for controlling the virus. The high evolutionary rate of SARS-CoV-2, usual for an RNA virus, has led to a number of mutations that appear to impact virus fitness (2). Genetic variants of SARS-CoV-2 can be classified into variants of concern (VOCs) and variants of interest (VOIs) (3). VOCs have been associated with an increased transmissibility and reduction in neutralization by natural or vaccine-derived antibodies and may cause more severe symptoms. VOCs share the N501Y change in the receptor binding domain (amino acids 319 to 541) of the spike glycoprotein (S) that provides a higher affinity toward the ACE2 host receptor (4, 5). These VOCs or lineages were originally detected in the United Kingdom (B.1.1.7), South Africa (B.1.351), and Brazil (B.1.1.28.1 or P.1). The VOIs have specific genetic markers (i.e., E484K) that might affect infectivity and immune response (6), and they comprise the B.1.536 and B.1.525 variants from New York and B.1.1.28.2 (P.2) from Brazil.

The research described in this study was performed in adherence to the Declaration of Helsinki; no specific authorization was required, because the activities were conducted as part of a routine virological surveillance (anonymously, without identification of patients) by the Uruguayan official Institution for Surveillance of Influenza and Other Respiratory Viruses of the Ministry of Public Health (DLSP-MSP).

The variants P.1 and P.2, which emerged in Brazil, have spread to other parts of South America (7, 8). Here, we describe the genomes of SARS-CoV-2 variants P.1 (SARS-CoV-2/human/URY/374/2021) and P.2 (SARS-CoV-2/human/URY/380/2021) detected in Uruguay.

Nasopharyngeal swab samples were collected in March 2021 in the Uruguayan Rivera department bordering Brazil and came from two symptomatic cases. The samples tested positive for SARS-CoV-2 using a standard quantitative PCR (qPCR) procedure (9); both patients had a threshold cycle (*C_T_*) value of <18. RNA was extracted with a QIAmp viral minikit (Qiagen, USA). Genome amplification was achieved using ARTIC 3 primers (https://artic.network/ncov-2019). First, cDNA strand analysis, Nextera DNA Flex library preparation, and 2 × 150-bp sequencing on an Illumina MiniSeq platform were performed following a previous report (10). Adapter/quality trimming and filtering of raw data were performed with BBDuk, and clean reads were mapped using Geneious Prime. Annotation and identification of nucleotide mutations were performed with Geneious software and with CoV-GLUE (http://cov-glue.cvr.gla.ac.uk/). Lineages refer to those assigned using the pangolin tool (https://cov-lineages.org). All tools were run with default parameters unless otherwise specified.

Sample SARS-CoV-2/human/URY/374/2021 (P.1) has a sequence length of 29,835 nucleotides (nt), 1,240,211 total reads, 3,920× mean coverage, and a 38.0% G+C content. Sample SARS-CoV-2/human/URY/380/2021 (P.2) has a sequence length of 29,858 nt, 1,007,202 total reads, 5,877× mean coverage, and a 37.9% G+C content. Their genome sequences lack the outermost nucleotides (<20 nt) of the 5′ and 3′ untranslated regions (UTRs), which are not usually sequenced with the ARTIC protocol.

The VOC P.1 genome is 99.89% identical to the Wuhan-Hu-1 reference genome but has several amino acid substitutions (Table 1). The S protein has 12 replacements, including 10 variant-specific substitutions (7). It also has a codon-aligned deletion (106 to 108) in *nsp6* that is considered a P.1 genetic signature (7). The VOI P.2 genome also has 99.89% identity to the reference genome but has only 4 replacements in the S protein, including the independently acquired E484K marker, and lacks indels (Table 1).

**TABLE 1:** Genetic changes of variants P.1 and P.2 from Uruguay compared to the Wuhan-Hu-1 SARS-CoV-2 reference sequence (GenBank accession number NC_045512)

Variant name	Genome region (position)	Codon change	Amino acid change (position)
P.1	ORF1ab-nsp3 (3827–3829)	TCA→TTA	S→L (370)
P.1	ORF1ab-nsp3 5648–5650	AAA→CAA	K→Q (977)
P.2	ORF1ab-nsp5 (10667–10669)	TTA→GTA	L→V (205)
P.1	ORF1ab-nsp6 (11288–11296)	Del (TCTGGTTTT)	SGF (106–108)
P.2	ORF1ab-nsp7 (12053–12055)	CTT→TTT	L→F (71)
P.1/P.2	ORF1ab-nsp-12 (14407–14409)	CCT→CTT	P→L (323)
P.1	ORF1ab-nsp-13 (17257–17259)	GAG→GAT	E→D (341)
P.1	S (21614–21617)	CTT→TTT	L→F (18)^*a*^
P.1	S (21620–21622)	ACC→AAC	T→N (20)^*a*^
P.1	S (21638–21640)	CCT→TCT	P→S (26)^*a*^
P.1	S (21974–21976)	GAT→TAT	D→Y (138)^*a*^
P.1	S (22130–22132)	AGG→AGT	R→S (190)^*a*^
P.1	S (22811–22813)	AAG→ACG	K→T (417)^*a*^
P.1/P.2	S (23012–23014)	GAA→AAA	E→K (484)^*a*^
P.1	S (23063–23065)	AAT→TAT	N→Y (501)^*a*^
P.1/P.2	S (23402–23404)	GAT→GGT	D→G (614)
P.1	S (23525–23527)	CAT→TAT	H→Y (655)^*a*^
P.1	S (24641–24643)	ACT→ATT	T→I (1027)^*a*^
P.1/P.2	S (25088–25090)	GTT→TTT	V→F (1176)
P.2	S (25247–25249)	ATG→ATT	M→I (1229)
P.1	ORF3a (26149–26151)	TCC→CCC	S→P (253)
P.2	M (26589–26561)	GTA→TTA	V→L (23)
P.1	ORF8 (28167–28169)	GAA→AAA	E→K (92)
P.1	Intergenic region (28263–28266)	Ins (AACA)
P.1	N (28515–28517)	CCA→CGA	P→R (80)
P.2	N (28632–28634)	GCT→TCT	A→S (119)
P.1/P.2	N (28844–28846)	AGG→AAA	R→K (203)
P.1/P.2	N (28887–28889)	GGA→CGA	G→R (204)
P.2	N (28997–28999)	ATG→ATT	M→I (234)

a P.1-specific substitutions on the spike (S) protein.

A significant increase in the numbers of cases and deaths has been occurring in Uruguay since March 2021, coinciding with the appearance and increase of P.1 and P.2 variants in the territory. The identification of variants with potentially new biological properties encourages the efforts of doing genomic surveillance to contribute to controlling the pandemic.

### Data availability.

These genome sequences were deposited in GenBank under accession numbers MW988204 (P.1, SARS-CoV-2/human/URY/374/2021) and MW988205 (P.2, SARS-CoV-2/human/URY/380/2021). The raw reads and metadata were deposited under the BioProject accession number PRJNA634396 and SRA accession numbers SRX10652818 (SARS-CoV-2/human/URY/374/2021) and SRX10652819 (SARS-CoV-2/human/URY/380/2021).
